# Plugging Small RNAs into the Network

**DOI:** 10.1128/mSystems.00422-20

**Published:** 2020-06-02

**Authors:** Lars Barquist

**Affiliations:** a Helmholtz Institute for RNA-based Infection Research (HIRI), Helmholtz Centre for Infection Research (HZI), Würzburg, Germany; b Faculty of Medicine, University of Würzburg, Würzburg, Germany

**Keywords:** network inference, ncRNA, sRNA

## Abstract

Small RNAs (sRNAs) have been discovered in every bacterium examined and have been shown to play important roles in the regulation of a diverse range of behaviors, from metabolism to infection. However, despite a wide range of available techniques for discovering and validating sRNA regulatory interactions, only a minority of these molecules have been well characterized. In part, this is due to the nature of posttranscriptional regulation: the activity of an sRNA depends on the state of the transcriptome as a whole, so characterization is best carried out under the conditions in which it is naturally active. In this issue of *mSystems*, Arrieta-Ortiz and colleagues (M. L. Arrieta-Ortiz, C. Hafemeister, B. Shuster, N. S. Baliga, et al., mSystems 5:e00057-20, 2020, https://doi.org/10.1128/mSystems.00057-20) present a network inference approach based on estimating sRNA activity across transcriptomic compendia. This shows promise not only for identifying new sRNA regulatory interactions but also for pinpointing the conditions in which these interactions occur, providing a new avenue toward functional characterization of sRNAs.

## COMMENTARY

Two decades after the first genome-wide screens showed small RNAs (sRNAs) to be a widespread feature of bacterial genomes, these short noncoding transcripts remain enigmatic. High-throughput RNA sequencing (RNA-seq)-based approaches now routinely find hundreds of potential sRNAs in any bacterium examined (1). Despite this, only on the order of dozens of sRNAs have been characterized in any detail, and the number with clearly defined physiological roles is even smaller—in fact, it remains unclear what fraction may even possess a function at all (2). While some characterized sRNAs have specialized functions, such as titrating regulatory RNA-binding proteins (3), the majority appear to effect their function through RNA-RNA interactions with target transcripts. Depending on the molecular details and protein factors recruited, these interactions can have a wide range of consequences, including activating translation (4) or affecting transcript termination (5), but the most common outcome appears to be repression of translation often with a concomitant decay of the target transcript. In the best-characterized bacterial systems, Escherichia coli and Salmonella enterica, many of these sRNAs appear to form regulatory networks centered on RNA-binding proteins that facilitate RNA interactions, most famously Hfq (6) and the more recently discovered ProQ (7).

The bottleneck in sRNA characterization is not a lack of techniques. A basic molecular toolkit for identifying and validating sRNA-target interactions was developed in the mid-2000s by several different groups (8), and includes techniques like sRNA pulse induction followed by transcriptomics to identify putative targets, reporter systems to validate *in vivo* interactions, and structural probing to map the details of base pairs formed in the interaction. Computational tools for predicting interactions based on thermodynamic models of RNA folding have also been long available and are useful for understanding individual interactions (9) but generally have too high a false-positive rate to be useful for genome-wide predictions (10), though methods incorporating interaction conservation (11) or experimental evidence (12) are beginning to improve this. More recently, approaches based on coupling pulldowns to high-throughput sequencing have been developed. These approaches include GRIL-seq (global small noncoding RNA target identification by ligation and sequencing) (13) and MAPS (MS2 affinity purification coupled with RNA sequencing) (14), which involve pulling down an sRNA of interest with or without ligation, and RIL-seq (RNA interaction by ligation and sequencing) (15), which relies on pulling down an RNA-binding protein and ligating the RNA species interacting on it. These methods can provide direct evidence for sRNA-target interactions, though they also often report tens to hundreds of potential interactions, and it remains unclear how many of these impact cell physiology.

Beyond any inherent limitations of the techniques available, there is a more fundamental reason that sRNAs are difficult to characterize, namely, the inherently epistatic nature of posttranscriptional regulation. It was recognized early on that sRNA-based regulation is dependent on the overall cellular state in a way that transcriptional regulators are not (16). Most obviously, an sRNA cannot regulate a transcript that is not expressed under the tested conditions. More subtly, artificially induced competition for central RNA-binding proteins can have dramatic global effects on sRNA-based regulation (17), and it is becoming clear that similar phenomena affect regulation under more ordinary conditions (18). Other factors, such as the condition-specific expression of sRNA sponges (19), may reduce the regulatory activity of sRNAs. There is even evidence of cross talk between the networks mediated by RNA-binding proteins (20), adding another layer of complexity. We are then left in a situation where we cannot easily determine sRNA function because we do not know the conditions it is relevant to, and we do not know the conditions it is relevant to because we do not know the function.

Arrieta-Ortiz and colleagues offer one way out of this conundrum (21), using network inference (NI). NI is a collection of techniques that aim to reconstruct an underlying regulatory network from large collections of transcriptomic data. These techniques range from methods that apply clustering or thresholding approaches to simple statistics like Pearson correlations or mutual information to systems of ordinary differential equations that attempt to provide a working dynamic model of regulatory interactions (22). One of the reasons these methods have not been more widely adopted is the lack of large transcriptomic atlases from which to derive networks, outside certain model organisms like E. coli. In fact, the mutual information-based context likelihood of relatedness (CLR) algorithm was applied to sRNA network prediction in E. coli almost a decade ago (23), with promising preliminary results. Now with the wide availability of RNA-seq making such compendia easier to produce (24–26), NI approaches are due for a revival.

The key innovation of Arrieta-Ortiz and colleagues’ method (21) is to account for the epistatic nature of sRNA regulation in applying NI. Building on their previous work with transcription factors (27), they first apply a dimensionality reduction technique called network component analysis (28) that uses an incomplete map of the regulatory architecture of the cell to derive sRNA activity scores across the input transcriptomic compendium accounting for known regulatory interactions. They show that these scores anticorrelate more strongly with the expression level of sRNA targets than the expression level of sRNAs themselves, as would be expected in the presence of potential confounding factors. They then show that using these activity scores in place of sRNA expression improves the recovery of known sRNA-target interactions not included in the prior network with both their own NI approach, Inferelator (29), and a hybrid approach based on CLR. With applications in E. coli, Pseudomonas aeruginosa, Bacillus subtilis, and Staphylococcus aureus using a variety of prior network architectures derived from noisy computational or high-throughput experimental approaches, they show that their method consistently reports sRNA-target interactions with independent experimental support. In a final proof of concept, the authors investigate the distribution of activity scores across conditions of the P. aeruginosa sRNA PrrF, recovering known activity in iron-limited, biofilm, and virulence conditions, and the S. aureus sRNA RsaE, suggesting a role in the response to antibiotic stress.

The work in this study clearly demonstrates the utility of NI approaches for generating hypotheses regarding sRNA function and identifying new potential target mRNAs, and importantly, helps to situate the sRNA in something approaching its natural regulatory context. With the continuing accumulation of transcriptomic data in public repositories, it is easy to imagine that in the not too distant future, NI will join molecular techniques, computational target prediction, and high-throughput sequencing-based approaches as a standard part of the sRNA biologist’s toolkit.

## References

[1] Barquist L, Vogel J. 2015. Accelerating discovery and functional analysis of small RNAs with new technologies. Annu Rev Genet 49:367–394. doi:10.1146/annurev-genet-112414-054804. 26473381

[2] Jose BR, Gardner PP, Barquist L. 2019. Transcriptional noise and exaptation as sources for bacterial sRNAs. Biochem Soc Trans 47:527–539. doi:10.1042/BST20180171. 30837318

[3] Romeo T, Babitzke P. 2018. Global regulation by CsrA and its RNA antagonists. Microbiol Spectr 6(2):RWR-0009-2017. doi:10.1128/microbiolspec.RWR-0009-2017. PMC5868435 29573256

[4] Fröhlich KS, Vogel J. 2009. Activation of gene expression by small RNA. Curr Opin Microbiol 12:674–682. doi:10.1016/j.mib.2009.09.009. 19880344

[5] Kavita K, de Mets F, Gottesman S. 2018. New aspects of RNA-based regulation by Hfq and its partner sRNAs. Curr Opin Microbiol 42:53–61. doi:10.1016/j.mib.2017.10.014. 29125938 PMC10367044

[6] Vogel J, Luisi BF. 2011. Hfq and its constellation of RNA. Nat Rev Microbiol 9:578–589. doi:10.1038/nrmicro2615. 21760622 PMC4615618

[7] Olejniczak M, Storz G. 2017. ProQ/FinO-domain proteins: another ubiquitous family of RNA matchmakers? Mol Microbiol 104:905–915. doi:10.1111/mmi.13679. 28370625 PMC5578414

[8] Sharma CM, Vogel J. 2009. Experimental approaches for the discovery and characterization of regulatory small RNA. Curr Opin Microbiol 12:536–546. doi:10.1016/j.mib.2009.07.006. 19758836

[9] Umu SU, Gardner PP. 2017. A comprehensive benchmark of RNA-RNA interaction prediction tools for all domains of life. Bioinformatics 33:988–996. doi:10.1093/bioinformatics/btw728. 27993777 PMC5408919

[10] Pain A, Ott A, Amine H, Rochat T, Bouloc P, Gautheret D. 2015. An assessment of bacterial small RNA target prediction programs. RNA Biol 12:509–513. doi:10.1080/15476286.2015.1020269. 25760244 PMC4615726

[11] Wright PR, Richter AS, Papenfort K, Mann M, Vogel J, Hess WR, Backofen R, Georg J. 2013. Comparative genomics boosts target prediction for bacterial small RNAs. Proc Natl Acad Sci U S A 110:E3487–E3496. doi:10.1073/pnas.1303248110. 23980183 PMC3773804

[12] Holmqvist E, Wright PR, Li L, Bischler T, Barquist L, Reinhardt R, Backofen R, Vogel J. 2016. Global RNA recognition patterns of post‐transcriptional regulators Hfq and CsrA revealed by UV crosslinking in vivo. EMBO J 35:991–1011. doi:10.15252/embj.201593360. 27044921 PMC5207318

[13] Han K, Tjaden B, Lory S. 2016. GRIL-seq provides a method for identifying direct targets of bacterial small regulatory RNA by in vivo proximity ligation. Nat Microbiol 2:16239. doi:10.1038/nmicrobiol.2016.239. 28005055 PMC5567838

[14] Lalaouna D, Carrier M-C, Semsey S, Brouard J-S, Wang J, Wade JT, Massé E. 2015. A 3′ external transcribed spacer in a tRNA transcript acts as a sponge for small RNAs to prevent transcriptional noise. Mol Cell 58:393–405. doi:10.1016/j.molcel.2015.03.013. 25891076

[15] Melamed S, Peer A, Faigenbaum-Romm R, Gatt YE, Reiss N, Bar A, Altuvia Y, Argaman L, Margalit H. 2016. Global mapping of small RNA-target interactions in bacteria. Mol Cell 63:884–897. doi:10.1016/j.molcel.2016.07.026. 27588604 PMC5145812

[16] Gottesman S. 2004. The small RNA regulators of Escherichia coli: roles and mechanisms. Annu Rev Microbiol 58:303–328. doi:10.1146/annurev.micro.58.030603.123841. 15487940

[17] Papenfort K, Said N, Welsink T, Lucchini S, Hinton JCD, Vogel J. 2009. Specific and pleiotropic patterns of mRNA regulation by ArcZ, a conserved, Hfq-dependent small RNA. Mol Microbiol 74:139–158. doi:10.1111/j.1365-2958.2009.06857.x. 19732340

[18] Faigenbaum-Romm R, Reich A, Gatt YE, Barsheshet M, Argaman L, Margalit H. 2020. Hierarchy in Hfq chaperon occupancy of small RNA targets plays a major role in their regulation. Cell Rep 30:3127–3138.e6. doi:10.1016/j.celrep.2020.02.016. 32130912 PMC7059120

[19] Azam MS, Vanderpool CK. 2015. Talk among yourselves: RNA sponges mediate cross talk between functionally related messenger RNAs. EMBO J 34:1436–1438. doi:10.15252/embj.201591492. 25916829 PMC4474520

[20] Melamed S, Adams PP, Zhang A, Zhang H, Storz G. 2020. RNA-RNA interactomes of ProQ and Hfq reveal overlapping and competing roles. Mol Cell 77:411–425.e7. doi:10.1016/j.molcel.2019.10.022. 31761494 PMC6980735

[21] Arrieta-Ortiz ML, Hafemeister C, Shuster B, Baliga NS, Bonneau R, Eichenberger P. 2020. Inference of bacterial small RNA regulatory networks and integration with transcription factor-driven regulatory networks. mSystems 5:e00057-20. doi:10.1128/mSystems.00057-20. 32487739 PMC8534726

[22] De Smet R, Marchal K. 2010. Advantages and limitations of current network inference methods. Nat Rev Microbiol 8:717–729. doi:10.1038/nrmicro2419. 20805835

[23] Modi SR, Camacho DM, Kohanski MA, Walker GC, Collins JJ. 2011. Functional characterization of bacterial sRNAs using a network biology approach. Proc Natl Acad Sci U S A 108:15522–15527. doi:10.1073/pnas.1104318108. 21876160 PMC3174649

[24] Kröger C, Colgan A, Srikumar S, Händler K, Sivasankaran SK, Hammarlöf DL, Canals R, Grissom JE, Conway T, Hokamp K, Hinton J. 2013. An infection-relevant transcriptomic compendium for *Salmonella enterica* serovar Typhimurium. Cell Host Microbe 14:683–695. doi:10.1016/j.chom.2013.11.010. 24331466

[25] Kröger C, MacKenzie KD, Alshabib EY, Kirzinger MWB, Suchan DM, Chao T-C, Akulova V, Miranda-CasoLuengo AA, Monzon VA, Conway T, Sivasankaran SK, Hinton JCD, Hokamp K, Cameron A. 2018. The primary transcriptome, small RNAs and regulation of antimicrobial resistance in Acinetobacter baumannii ATCC 17978. Nucleic Acids Res 46:9684–9698. doi:10.1093/nar/gky603. 29986115 PMC6182133

[26] Warrier I, Ram-Mohan N, Zhu Z, Hazery A, Echlin H, Rosch J, Meyer MM, van Opijnen T. 2018. The transcriptional landscape of Streptococcus pneumoniae TIGR4 reveals a complex operon architecture and abundant riboregulation critical for growth and virulence. PLoS Pathog 14:e1007461. doi:10.1371/journal.ppat.1007461. 30517198 PMC6296669

[27] Arrieta-Ortiz ML, Hafemeister C, Bate AR, Chu T, Greenfield A, Shuster B, Barry SN, Gallitto M, Liu B, Kacmarczyk T, Santoriello F, Chen J, Rodrigues CDA, Sato T, Rudner DZ, Driks A, Bonneau R, Eichenberger P. 2015. An experimentally supported model of the Bacillus subtilis global transcriptional regulatory network. Mol Syst Biol 11:839. doi:10.15252/msb.20156236. 26577401 PMC4670728

[28] Liao JC, Boscolo R, Yang Y-L, Tran LM, Sabatti C, Roychowdhury VP. 2003. Network component analysis: reconstruction of regulatory signals in biological systems. Proc Natl Acad Sci U S A 100:15522–15527. doi:10.1073/pnas.2136632100. 14673099 PMC307600

[29] Bonneau R, Reiss DJ, Shannon P, Facciotti M, Hood L, Baliga NS, Thorsson V. 2006. The Inferelator: an algorithm for learning parsimonious regulatory networks from systems-biology data sets de novo. Genome Biol 7:R36. doi:10.1186/gb-2006-7-5-r36. 16686963 PMC1779511

